# Canakinumab might be Protective against Severe COVID-19 for Patients with Autoinflammatory Disorders

**DOI:** 10.31138/mjr.33.2.237

**Published:** 2022-06-30

**Authors:** Arif Alkan, Serdal Ugurlu

**Affiliations:** 1 Cerrahpasa Medical Faculty, University of Istanbul-Cerrahpasa, Istanbul, Turkey,; 2 Division of Rheumatology, Department of Internal Medicine, Cerrahpasa Medical Faculty, University of Istanbul-Cerrahpasa, Istanbul, Turkey

**Keywords:** Canakinumab, Covid-19, Autoinflammatory disease, Familial Mediterranean Fever

## INTRODUCTION

Coronavirus infection (COVID-19) causes systemic inflammation with a predilection to pulmonary tissue and in severe cases, acute respiratory distress syndrome (ARDS) occurs as a complication. Complications of the disease (pneumonia, ARDS, etc.) are believed to be caused by uncontrolled inflammatory responses.^1^ Familial Mediterranean fever (FMF) is an autoinflammatory disease characterised by recurrent attacks of fever and serositis. The dysregulated activity of the innate immune system causes high interleukin-1 (IL-1) levels. Prophylactic colchicine treatment is sufficient for 95% of the cases, yet in the cases of colchicine resistance, IL-1 antagonists are required (anakinra, canakinumab, rilonacept).^2^ Systemic juvenile idiopathic arthritis (sJIA) and adult-onset still disease (AOSD) are multi-genetic autoinflammatory disorders where IL-1 plays a major part. Both diseases are characterised by spiking fever and arthritis. While the mainstay of the treatment is glucocorticoids IL-1 antagonists are effective as both treatment of resistant disease and steroid-sparing agents.^3,4^ The effectiveness of anakinra on COVID-19 infection has been reported in a few studies.^5–7^ Canakinumab was investigated for myocarditis in COVID-19 patients in one study^8^ and canakinumab was associated with better outcomes in another.^9^ Herein we report on five cases of COVID-19 infection in patients with inflammatory disorders (3 FMF,1 AOSD,1 sJIA) who contracted the infection while being treated with canakinumab.

## METHODS

After recovery from the infection, detailed history and available test results were obtained. Written informed consent was obtained from the patients. Ethical board approval was not obtained as our institution does not require ethical board approval for case series.

## CASES

### Patient 1

A 23-year-old female patient presented to the emergency unit with a chief complaint of a productive cough. Her past medical history included FMF for 18 years and ulcerative colitis (UC) for 8 years. She had homozygous M694V mutation. She was stable under colchicine 2mg/day, canakinumab 150mg/8 weeks for FMF, and mesalazine 1600mg/day for UC. Her polymerase chain reaction (PCR) test was positive for COVID-19. Her chest X-ray was normal. The laboratory investigations at the time of admission and discharge are shown in **Table 1**. She was admitted to the inpatient unit due to her history of immunosuppressive treatment, and was treated with hydroxychloroquine (HCQ) 400mg/day, enoxaparin 6000 IU/day for 8 days and she continued her previous medication. During her illness, she did not develop any complications (pneumonia, sepsis, disseminated intravascular coagulation). She had one episode of abdominal pain attributable to FMF (she did not have diarrhoea, tenesmus). She was discharged eight days later from the hospital following two consecutive negative PCR results and the resolution of her symptoms. At her last visit, both of her diseases were in remission.

**Table 1.** Laboratory investigations of the patients.

	**Patient 1 Prior to Admission**	**Patient 1 After Discharge**	**Patient 2**	**Patient 3**	**Patient 4**	**Patient 5**
Leukocytes (x10^3^/mm^3^)	6.3	6.6	3.89	-	-	-
Neutrophils (x10^3^/mm^3^)	2.9	2.8	1.77	-	-	-
Lymphocytes (x10^3^/mm^3^)	2.7	3.4	1.88	-	-	-
C-Reactive Protein (mg/L)	12.9	15.9	6.2	-	-	-
Ferritin (ng/mL)	62.7	7.8	29.6	-	-	-
D-Dimer (ng/mL)	1640	170	<200	-	-	-
Procalcitonin (ng/mL)	0.03	0.03	-	-	-	-
Troponin I (ng/mL)	0.64	1	-	-	-	-
Oxygen Saturation	Between 96–98% during hospital stay on room air	Between 96–98% during hospital stay on room air	-	-	-	-
Fibrinogen (g/L)	1.68	2.98	-	-	-	-
PaO2/FiO2	>400	>400	>400	>400	>400	>400

### Patient 2

A 32-year-old female patient presented with throat pain, headache, and sneezing. Her past medical history included FMF for 28 years and hypothyroidism. She had a homozygous M694V mutation. She was stable under colchicine 1mg/day, canakinumab 150mg/4 weeks for FMF, and 50 mcg/day levothyroxine for hypothyroidism. She underwent a PCR test because her husband tested positive for COVID-19. Her thorax CT scan was normal and blood workup revealed mild leukopenia and mild elevation of CRP (**Table 1**). She was prescribed favipiravir for outpatient treatment and continued to her previous medications. Ten days after the PCR test came back negative and her symptoms were resolved by that time. At her last visit, her symptoms were in remission.

### Patient 3

A 20-year-old male patient presented with mild back pain and loss of smell and taste sensations. His medical history included systemic juvenile idiopathic arthritis for 11 years. He was stable under colchicine 1mg/day and canakinumab 150mg/4 weeks. His PCR test was positive for COVID-19. He was prescribed favipiravir for outpatient treatment and continued with his previous medication. His back pain, loss of taste, and smell resolved after 2, 3, and 7 days respectively. After 10 days in quarantine, he was deemed to have recovered. At his last visit, his symptoms were in remission.

### Patient 4

A 30-year-old female patient presented with a non-productive cough and headache. Her medical history included adult-onset still disease. She was stable under prednisolone 5mg/day, HCQ 200 mg/day, methotrexate 20mg/week, and canakinumab 150mg/4 weeks. Her PCR test was positive for COVID-19. She continued her previous medication with no other treatment applied. She was deemed to have recovered after her quarantine of 14 days. At her last visit, her disease was stable.

### Patient 5

A 38-year-old female patient was diagnosed with FMF for 20 years and amyloidosis. She had a homozygous M694V mutation She was under treatment with colchicine 2mg/day and canakinumab 150mg/4 weeks. Her PCR test for COVID-19 was positive despite not having symptoms. Later she developed a loss of smell and taste sensation which lasted for three days. She was treated with favipiravir. After quarantine of 14 days, she was deemed to have recovered. At her last visit, she had minimal pleural effusion and related chest pain. We think this is because of delayed administration of canakinumab due to her quarantine. Canakinumab dose was repeated. She also had mild proteinuria (78mg/day) related to AA amyloidosis.

## RESULTS

All the patients in this case series presented with mild upper respiratory tract infection symptoms or/and loss of smell and taste sensation. None of the patients developed pneumonia or other complications of the disease (ARDS, sepsis, etc.). All the patients were stable regarding their autoinflammatory disease. Only Patient 1 was admitted to hospital for monitoring purposes rather than the indication for in-hospital treatment. All of the patients recovered and none of the patients have persistent symptoms.

## DISCUSSION

COVID-19 binds to ACE-2 receptors in target cells. The genome of SARS-COV2 encodes viroporin protein 3a. This protein can directly activate NLPR3 (NOD-like receptor family pyrin domain containing 3). Furthermore, failure to clear the virus causes continuous NLPR3 activation. NLPR3 inflammasome activates caspase-1 which ultimately leads to IL-1β secretion.^10,11^ IL-1β promotes the production of transforming growth factor (TGF)-β, induces the secretion of neutrophil chemokines causing neutrophil influx to the lung, promotes alveolar epithelial cell death, and enhances the production of platelet-derived growth factor (PDGF). The result of this pathway is pulmonary fibrosis. Therefore IL-1β over-secretion is one of the important mechanisms of lung injury in COVID-19.^12^ FMF is caused by mutations in the MEFV gene which encodes pyrin/marenostrin. Pyrin interacts with ASC (Apoptosis-associated speck-like protein containing a CARD)leading to caspase-1 activation. Activation of caspase-1 leads to the processing of pro-IL-1β followed by activation and secretion of IL-1β.^2^ This mechanism is like cytokine overproduction seen in COVID-19. Therefore, it is expected to see higher rates of hyperinflammation and complications of COVID-19 in these patients who are already predisposed to inflammation. Patients 1,2,5 had homozygous M694V mutation which is one of the most severe mutations for disease activity itself but the unknown significance for COVID-19 infection probably being protective against acquiring the infection but aggravating factor for hyperinflammation.^2,13^ Patient one also had ulcerative colitis but it is associated with a higher risk for acquiring the infection despite no increased risk for severe infection as she was in the low-risk group.^14^ sJIA and AOSD are autoinflammatory diseases of unknown aetiology. IL-1 plays a substantial part in disease pathogenesis shown by the dramatic response to IL-1 antagonists. Furthermore, Macrophage activation syndrome (MAS), which is a well-known complication of both sJIA and AOSD, is similar to the hyperinflammation seen in COVID-19. IL-1 has a dominant role in both MAS and hyperinflammation. Patients 3 and 4 even had an increased tendency to develop hyperinflammation because of underlying inflammatory diseases had mild COVID-19 infection.^3–4,15–16^ Despite underlying inflammatory conditions and amyloidosis in patient five they all had mild disease. The data on canakinumab usage during COVID-19 infection is scarce although there are studies reporting the efficacy of canakinumab for COVID-19 treatment.^8, 9,17^ Canakinumab treatment might prevent COVID-19 complications and might be, although it is not possible to draw definitive conclusions from five patients and existing data is about treatment rather than prevention, associated with milder disease course. Thus, we suggest patients who are using canakinumab continue their treatment during COVID-19 infection.

## LIMITATIONS

Chest X-ray of Patient 1 is unavailable for publication as we do not have the image.

All of the patients were diagnosed and recovered within the dosing interval of canakinumab; the canakinumab was not repeated.

None of the patients were admitted to our centre for COVID-19; therefore, their management does not represent our approach.

In patients 3,4 and 5, no other test than PCR for SARSCoV-2-RNA was performed.

## Data Availability

The data underlying this article is available in the article and its online supplementary material.

## DECLARATION

Informed consent was obtained from the patients.
